# Building capacity for translational simulation: the role of faculty development

**DOI:** 10.1186/s41077-026-00470-7

**Published:** 2026-08-10

**Authors:** Victoria Brazil, Ellen Davies, Ben Symon, Adam Montagu, Jane Cichero, Nancy Sadka, Eve Purdy

**Affiliations:** 1 Faculty of Health Sciences and Medicine, Bond University, Gold Coast, QLD Australia; 2 Adelaide Health Simulation, College of Health, Adelaide University, Adelaide, SA Australia; 3 Mater Health Simulation Service, Mater Health South Brisbane, Brisbane, QLD Australia; 4 Sydney Childrens Hospital Network Sydney, Sydney, NSW Australia; 5 Western Health Footscray, Melbourne, VIC Australia

**Keywords:** Translational simulation, Faculty development, Healthcare simulation

## Abstract

**Introduction:**

Translational simulation is now established as a conceptual framing for aligning simulation with healthcare quality and safety priorities, but the development of organisational capacity for this work remains poorly described. We explored how translational simulation programs build capacity, with a specific focus on the role of formal faculty development.

**Methods:**

We used a constructivist qualitative approach informed by collaborative autoethnographic principles to explore how translational simulation programs build capacity through program-level decisions and faculty development strategies. Data were generated through a recursive, multi-stage narrative process, including structured written accounts from leaders of three Australian simulation programs and a narrative from the coordinator of the group-based Applied Translational Simulation course. Narratives were iteratively revised in response to written prompts to deepen description and interpretation. Data were analysed using reflexive thematic analysis, with concepts and themes developed through paired review, whole-team discussion, and re-examination of the complete dataset.

**Results:**

Five themes were identified: strategy and leadership drive capacity building; program context shapes approach; translational simulation capacity is integrated, not isolated; trigger events create motivation and opportunity; and building capacity is relational. Capacity is developed through doing translational simulation work, supported by mentorship, projects, advocacy, and shared language, with formal faculty development providing structure and legitimacy.

**Conclusions:**

Faculty development contributes most when embedded within broader institutional conditions that support translational simulation practice.

**Supplementary Information:**

The online version contains supplementary material available at https://doi.org/10.1186/s41077-026-00470-7.

## Introduction

As healthcare organisations embrace simulation to improve quality and safety, the challenge is no longer simply how to design individual simulation activities, but how to build programmatic capacity for simulation. Translational simulation provides a conceptual framing for this shift [1, 2], moving simulation beyond educational paradigms to focus on directly exploring and improving healthcare systems. The *purpose* and *process* of translational (or ‘systems-focused’) simulation are increasingly well understood [2–4], but the enablers of translational simulation at the program or institution level are underexplored. Faculty development is a potential enabler, but its effects are inseparable from the organisational and relational contexts in which it is delivered [5]. A deeper understanding of how faculty development strategies interact with contextual factors will provide helpful guidance for programs seeking to build and sustain translational simulation capacity.

Faculty development is an established element in most *educationally* focused simulation programs, supported by a mature literature describing common content areas [6], formalised development pathways [7, 8], and an emerging role of professional societies [9]. By contrast, the literature describing faculty development for *translational* simulation is sparse. Existing work suggests that traditional simulation faculty development does not fully prepare faculty for the procedural, strategic, and operational demands of translational simulation [10]. Members of our author team previously found that faculty development practices in this area are often diverse, informal, and strongly shaped by local context [5]. Content areas for faculty development in the programs we studied included domains familiar to simulation-based education (scenario design, debriefing, psychological safety etc.) but also content specific to the translational context (e.g. quality improvement (QI) methodology, human factors and systems science). Collaboration beyond the historical boundaries of the healthcare simulation community was an enabler across themes in the study [5]. Given the powerful influence of context and collaboration, faculty development strategies are likely both an antecedent and an outcome of successful translational simulation capacity building.

A related body of literature has begun to describe how translational simulation programs are built, embedded, and sustained [11–15]. Across this work, leadership [13], governance [11], resources, partnerships [11, 14, 15], and change management [12] emerge as important enablers. Faculty development is recognised within this broader picture but less is known about how it interacts with these program-level factors to support capacity building.

To address this gap, we explored the motivations and experience of three simulation programs in Australia who have participated in an institutional approach to faculty development for translational simulation. We sought to build upon earlier exploration of faculty development activities within simulation programs, by asking the research question: how and why do translational simulation programs *build capacity*, through program level decisions, and what role does faculty development play in that process? 

## Methods

### Study context

Following our prior exploration of the faculty development practices of translational simulation programs [5], some of our author team (VB, EP, BS) developed a formal program in translational simulation at Bond University, Gold Coast, Australia. The program is offered in two formats: as a 12-week subject for individual enrolment, and as an 8-week microcredential program (Applied Translational Simulation (ATS)) in which organisations can enrol a group of their staff to undertake the program together. The educational content of the program consists of six modules, informed by the study findings [5] and personal experience:

1. Introduction to translational simulation
2. Process for translational simulation (methods and tools)
3. Quality improvement and patient safety
4. Human factors and healthcare spaces
5. Teams and organisational culture
6. Capstone module.

Assessment requires submission of a translational simulation project relevant to the participant’s local context. The course has been delivered to eight institutions in Australia and the United Kingdom over 2024-6.

**Fig. 1:**
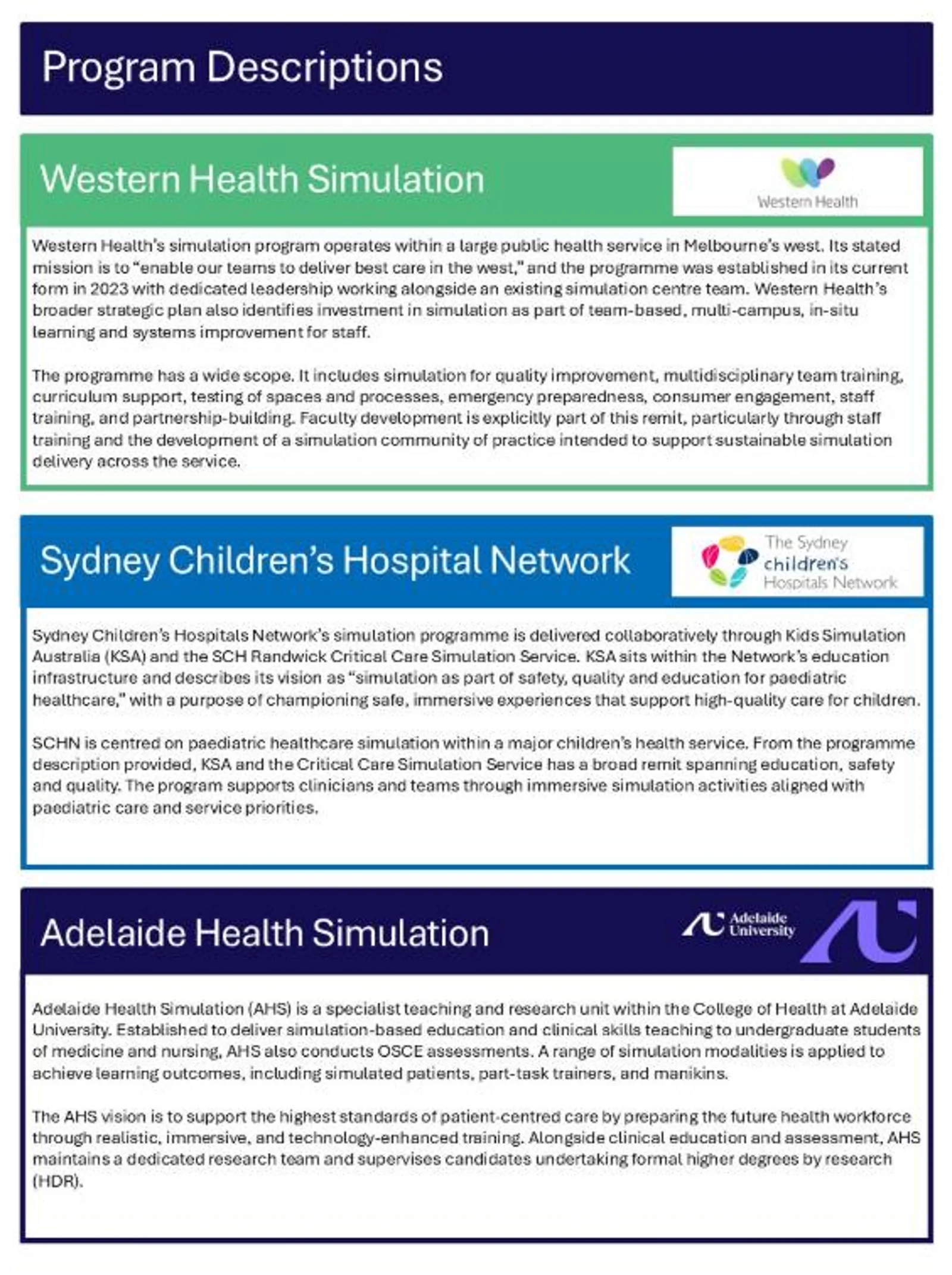
Program descriptions

Our author team was formed through relationships developed during delivery of the ATS microcredential in 2024 and 2025. VB, EP and BS are Bond University faculty for the ATS program and bring perspectives and experience from their other roles as translational simulation practitioners within their health service employment. In delivering multiple iterations of the course we became acutely aware of the variation in translational simulation program contexts, and how that affected the ability to translate faculty development activities into practice. As we sought to continuously refine and improve our ATS course, we were interested in better understanding that relationship between context and faculty development. JC, ED, AM and NS are simulation program leaders who led their teams to participate in the ATS program in 2024/ 2025. To provide further context, a description of these three programs is offered in Fig. 1. JC, AM and ED have experience as nurses, simulation educators and researchers. NS is an emergency physician and simulation leader. These roles and positioning, and our reflexivity, are reflected upon further in subsequent sections where relevant.

### Conceptual orientation

We adopted a constructivist orientation, recognising that our study questions required interpretation and co-construction of meaning from situated accounts of practice. The study was designed as a qualitative inquiry informed by collaborative autoethnographic principles [16–18], including the use of researchers’ own experiences as a source of data, and collaborative interpretation to generate meaning. Rather than claiming a full collaborative autoethnographic design, we drew on these principles to support a structured and reflexive examination of how translational simulation program leaders and course faculty understood capacity building through participation in ATS. We conceptualised the inquiry as a polylogue [19], in which multiple voices, roles and institutional perspectives were treated as an analytic resource [17]. Rather than seeking a singular authoritative account, we used recursive narrative writing, questioning and collective interpretation to explore how faculty development, workplace context and program strategy interacted.

Our data collection and analysis are sensitised by two faculty development frameworks developed within health professions education. Steinert’s approach considers faculty development activities on two axes: from formal to informal; and the context for learning, from individual to group [20]. The ATS program was clearly situated within the formal, group quadrant in this model. The three simulation programs explored in this study also engage in faculty development across the two axes, including formal and informal learning by individuals and groups through courses, workshops, coaching, mentoring and journal clubs. Members of the author team have also participated in, and delivered, faculty development activities for healthcare simulation across these spectra. In the second framework, O’Sullivan and Irby suggest that faculty development is embedded in two communities of practice - the faculty development community and the workplace community [21]. They further contend that bringing about desired change requires the interaction of four primary components (facilitator, participants, context, and program) with their associated processes (mentoring and coaching; relationships and networks; organizations, systems, and cultures; and tasks and activities) [21] These nuanced inter-relationships were recognised by the author team who each straddled roles within simulation, faculty development and workplace communities of practice. The Steinert and O’Sullivan frameworks informed our prompting questions and supported interpretation, rather than an explicit coding structure.

### Data collection

Data were generated through a recursive, multi-stage, written narrative process. Program leaders (NS, AM and JC) each wrote a structured account of their motivations for and experience in participating in the ATS micro credential. They were asked to recount in detail their simulation program activities, including the relative balance of educational and translational work, and contextual issues that influenced their program mission, scope, human resources and operational strategy. Their structured reflection included specific information on faculty development activities and opportunities for their staff. Prompting questions (supplementary material 1) for these written accounts were developed by VB and EP, sensitised by the Steinert [20] and O’ Sullivan [21] models, and reviewed by the author team. In parallel, the course leader (VB) wrote a narrative on the rationale for offering the Applied Translational Simulation subject as a group-based faculty development offering, including reflection on the pedagogical choices made with respect to content and process of subject delivery.

These first-round narratives were then then read by ED, who provided written prompting questions to elicit further depth, clarification and nuance. The original authors revised and elaborated their narratives in response. This recursive process produced a final dataset comprising three elaborated program leader narratives and one faculty narrative from the subject coordinator.

### Data analysis

The narratives were analysed using reflexive thematic analysis [22]. This approach aligned with our constructivist and collaborative orientation because it treats researcher subjectivity as a resource for interpretation and understands meaning as situated. Our aim was not to evaluate the effectiveness of the ATS program itself, but to use leaders’ experiences of the program as an entry point for examining a broader issue: how translational simulation programs make decisions about capacity building and faculty development. We recognised that the author team included both faculty who designed and delivered the ATS program and program leaders who had undertaken it. This created both insight and tension: shared language and trust enabled depth, while pre-existing relationships inevitably influenced what was noticed, asked and interpreted. We treated these dynamics as part of the analytic context rather than as bias to be eliminated.

Practically, the data analysis process occurred in stages. Firstly, all authors familiarised themselves with the data set, and undertook initial independent analysis. Authors then met as pairs to consider their initial reflections on the data set and identify concepts relevant to answering the study questions. Each pair comprised a program leader and faculty member, as contrasting perspectives. These findings (in written and verbal form) were then brought to a whole-team discussion, with further discussion and refinement of concepts and ideas seen in the data. Draft themes and sub themes were developed through this iterative process. VB and JC then re-reviewed the complete data set deductively, using the draft themes, to consider whether they were reflective of the data, and to identify any missing concepts. The final version of themes and illustrative quotations were considered and agreed by the whole author team.

## Results

We identified five themes in our exploration of how and why translational simulation programs build capacity, through program level decisions, combined with faculty development strategies: 1) Strategy and leadership drive translational simulation capacity building, 2) Program context shapes capacity building, 3) Translational simulation capacity is integrated, not isolated, 4) Trigger events create motivation and opportunity, and 5) Building capacity is relational. Translational simulation capacity was built through doing the work, supported by mentorship, projects, and ongoing advocacy. This capacity was supported and legitimised by formal faculty development. The five themes are described in this section, together with subthemes where relevant, and illustrative quotations drawn from the data. Programs are identified by their initials: SCHN (Sydney Children’s Hospital Network), WH (Western Health), and AHS (Adelaide Health Simulation).

### Strategy and leadership drive translational simulation capacity building

Although strategies, mission and scope of the simulation programs varied, they were clearly articulated by program leaders. This clarity, and the leaders that articulate and drive it, created conditions in which capacity building could occur and in which faculty development was more likely to be successful. We identified three sub-themes as we explored this issue in depth. 

#### Varied, but clear missions, scope and purpose

All narratives demonstrated impressive clarity about their programs overall, and specifically why translational simulation mattered in their setting. WH and SCHN had imminent new hospital builds. WH used translational simulation for ongoing service improvement needs, including hospital accreditation, transition to electronic medical record, supporting rapid response system roll-out, stroke patient pathway improvement, and an operating theatre utilisation initiative. AHS was looking to undertake translational simulation consultancy work, and to influence future clinician simulation leaders.

This clarity extended from high level mission statements to more granular operational strategy and was responsive to respective organisational priorities. Translational simulation focus was variable – from most of the work in one narrative to less than 10% in another – but the rationale for these choices was clear.

#### Leaders motivate and navigate contextual constraints

Leaders played a central role in building capacity through navigating organisational structures and cultures and aligning their programs with organisational priorities. Programs repeatedly faced limited human resources, fragile funding, workforce turnover, competing clinical demands, and variable executive buy-in. Leaders were negotiators of capacity growth within operational realities.

“*Understanding health service structure*,* reporting*,* governance*,* executive roles and ‘key influencers and decision makers’ is vital to inform* [translational simulation] *priorities and direct recommendations to the correct committee*,* executive*,* etc…”* (Simulation Program leader, AHS)

Recognising the critical role of leaders also unveiled a risk: the fragility of translational simulation endeavours due to reliance on champions to sustain and grow. This was in turn a motivator for those leaders to build capacity – through formal organisational systems, relationships and faculty development.

#### Advocacy and demonstrating value

Leaders recognised that building capacity for simulation required ongoing advocacy and demonstration of value for the organisation, and that this advocacy was needed from more than leaders alone. For translational simulation, a shared concept and shared language was perceived to support that advocacy, as was consistent methodology. The ATS course supported consistency and shared language among simulation team members, which in turn became familiar, trustworthy (and therefore fundable), to organisational executives.

*“The team members who attended the* [translational simulation] *course did all go on to lead [translational simulation]*,* …. what we have seen is them advocating in their own context for the role of simulation and them being able to champion the use of [translational simulation] appropriately.”* (Simulation Program leader, WH)

### Program context shapes capacity building approach

Program context shaped whether, how much, and what kind of faculty development was undertaken, and how that was integrated into broader capacity building initiatives. This was true for both translational and educationally focused simulation.

“*This understanding [of context] also informs the building of relationships and alignment between the simulation team and organisational priorities and goals. In order for us to influence our organisation we must understand its context and structure.*” (Simulation Program leader, WH)

Faculty development was perceived as developing knowledge and skills, while ‘building capacity’ was understood as more wide ranging, extending to what program faculty do with their knowledge and skills, leveraged (or not) by tools, systems and processes, and strategy. Three subthemes were identified and are described in this section.

#### Governance structures influence the approach

Governance arrangements were a crucial influence on how easily translational simulation capacity was built. This influence stemmed from governance within the programs themselves, and the positioning of the programs within the broader governance structure of their educational or health service institutions. Program alignment to quality and safety governance within a health service context supported clear direction and practical support for translational simulation work. When simulation activity and outputs connected to operational decision-making this became a reinforcing loop.

*“The Governance structure for* [translational simulation] *recommendations has been designed and integrated within Western Health governance and [business as usual] systems. We did not want to create separate processes for [translational simulation] recommendations.”* (Simulation Program leader, WH)

By contrast, translational simulation capacity was understandably prioritised lower when simulation programs were situated within educational frameworks and institutions. In these cases, organisations were often beneficiaries rather than active owners of translational simulation capacity building efforts, with simulation program leaders attuned to this context.

*“The most significant barrier is our context. AHS’s primary focus is undergraduate education in a university setting*,* and by necessity*,* we allocate most of our time and resources to this endeavour.”* (Simulation Program leader, AHS)

#### Value proposition perceived variably

Organisational priorities are shaped by context, funding models and missions, and this was, in turn, reflected in the support given to building capacity within the simulation programs in our study. The mere fact that these programs had funding to enrol their teams in the ATS course meant that value was placed on faculty development.

*“We believe the most important asset at AHS is the individuals who make up our team. Professional development is crucial to success*,* staff engagement*,* motivation and recognition of our high-performing team members.”* (Simulation Program leader, AHS)

However, the extent to which this support was extended to broader capacity building initiatives was dependent on the extent to which executives were convinced as to the value of translational simulation more broadly. This subtheme was closely connected to the advocacy role previously described.

*“[one] barrier is that our local health networks do not yet fully appreciate the value and application of translational simulation for quality improvement and system performance. To further build capacity*,* we need to be engaging in this work to provide faculty both experience and feedback.”* (Simulation Program leader, AHS)

#### Well-developed operational frameworks enable capacity

While faculty development grows individual skills, capacity building changes what the organisation can do with those skills. Hence, capacity building required tools, systems, staffing, resources, operational management, and organisational embedding. Programs had varying approaches to how they organised their translational simulation work, and varying levels of consistency and refinement of their tools and methodologies.

“*We have 2 arms … [of translational simulation] – regular projects which will remain ongoing and responsive projects (responsive to organisational needs – generally have a start and finish)”* (Simulation Program leader. WH)

Programs leaders were creative in leveraging resources that already existed - for example, simulation fellows on rotation from health services, clinician relationships and clinical networks - to build their operational capacity.

### Translational simulation capacity is integrated, not isolated

Translational simulation was layered to varying extents into existing simulation and education programs. This occurred through communities of practice, educator networks, routine simulation activity, and existing faculty development structures. This required as attention to the balance of this integration, as well as navigating tensions in the skills sets being built through faculty development, as illustrated in two subthemes.

#### Conceptual reframing: synergy and tension

Traditional simulation-based education experience and training had given faculty wide and deep capabilities: educational principles, attention to psychological safety, and skills in scenario design, small group facilitation and debriefing. Many of these were helpful for their emerging roles in translational simulation but could also be a barrier to embracing mindset shifts, systems thinking, and translational expertise. Program leaders recognised challenges in trying to help faculty embrace the breadth of conceptual foundations within translational simulation.

*“… safety science concepts*,* quality improvement methodology*,* governance systems around translational simulation*,* human factors*,* Safety II*,* implementation science*,* design thinking and complexity theory…. where to go to learn enough about these key areas without having to study each topic in isolation and in too much depth”* (Simulation Program leader, WH)

Translational simulation capacity building, including but not limited to faculty development, involved building from and upon simulation based educational mindsets, but also letting go. The ATS course was perceived to support *unfreezing moments* and *re-freezing* opportunities for those with education background.

*“[the team] would spend half their time in ‘sim educator’ mindset*,* doing great work with individuals and teams in training. But then we’d be invited… to join quality and safety issues with our ‘sim hats’*,* and the team found it hard to re-orientate their approaches to sim design*,* delivery and debriefing.”* (ATS Course coordinator)

#### Formal faculty development confers legitimacy (while building skills and shaping perspectives)

Participation in a recognised course functioned symbolically as credentialing or permission, legitimising translational simulation as a serious, defensible methodology within organisations.

“*We also saw this as an opportunity to showcase the value of simulation to many more clinicians and to executives & patient safety pillars of the organisation who in the main still viewed simulation just as an educational tool.*” (Simulation Program leader, SCHN)

Delivery from an external, tertiary institution validated translational simulation as modality that could be adopted in various contexts. This was a clear and understandable investment for organisations (course fees), that in turn fostered engagement in outcomes. Requiring project design as the assessment within the ATS course supported transfer to practice, but was insufficient to change practice without peer and program support for translational simulation work.

*“once you’ve outlaid money to buy a course*,* then you (exec*,* leadership*,* whomever foots the bill) want some ROI on that so you’re more likely to support the kind of projects that the course participants come up with.”* (ATS Course coordinator)

### Trigger events create motivation and opportunity

Capacity building frequently followed organisational triggers. Hospital re-design, commercial opportunities, organisational change, and partnership opportunities created impetus for building translational simulation capacity and for formal faculty development. This sense of urgency unlocked resources at higher levels, and enabled collaboration beyond the usual boundaries for simulation teams.

*“We had a short timeframe to bring staff on board as the move date came into view. We recognised the existing simulation services at SCHN could not possibly support what was needed to test and orientate the new areas and so had to come up with a practical ‘just in time’ solution”* (Simulation Program leader, SCHN)

However, programmes were not just *responsive* to organisational changes, but also proactive in identifying opportunities for new work or even revenue generation.

*“Formal [translational simulation] education was a requisite for engaging authentically in translational simulation consultancy work” (Simulation Program leader*,* AHS)*

Missing from any of our programs was a tight coupling between quality and safety monitoring and translational simulation work plans. However in one program, patient safety incidents were served as emergent ‘trigger events’, and motivation for change and improvement.

Trigger events also created a mix of ‘organisational emotions’ – opportunity, excitement, anxiety – that were motivators for the kind of team and organisational learning that translational simulation supports.

### Building capacity is relational

People were central to program leaders’ thinking about building capacity and the role of faculty development within that wider goal. The programs in our study already had strong and effective faculty teams prior to their participation in the ATS course.

#### Building multidisciplinary teams

Capacity grows when translational simulation is no longer the domain of the core simulation team alone. Simulation programs intentionally connected clinicians, educators, quality and safety staff, operational leaders, and health service executives to achieve their change management or improvement goals.

*“…bring in faculty with the more specialised skills e.g. process mapping*,* physical design & engineering*,* implementation science*,* lean six sigma*,* return on investment etc. So*,* partnering with others in the organisation or outside the organisation … – we can’t all know everything.* (Simulation Program leader, SCHN)

Courses - including the ATS program and in house faculty development - and working on translational simulation projects served as mechanisms to build these relationships.

“*… these folks [QI / project management/ organisational development/ patient safety people] had much less ‘unfreezing’ to do …And their participation was GOLD for the building capacity goal – the relationships and collaboration built with the ‘sim people’ at their institution seems to have been very valuable.”* (ATS Course coordinator)

These relational outcomes were also perceived to enable succession planning and broaden the leadership base. Faculty development nurtured local champions and demonstrated that they were valued.

#### Communities of practice for learning and work

Capacity for translational simulation was built through existing and emerging communities of practice (CoP), with recognisable mutual engagement, joint enterprise and shared repertoire characteristic of CoPs [23].

“*Our entire academic team and one simulation technician completed the ATS course. This resulted in the faculty simultaneously acquiring a common and shared understanding of translational simulation and its purpose. This shared understanding alone is a significant foundation for future translational simulation work.”* (Simulation Program leader, AHS)

Apprenticeship and mentorship were central to faculty development and building capacity in all programs, and likely a stronger contributor than formal faculty development. Courses provided foundational knowledge, but capacity developed through doing projects, that allowed knowledge to be applied and refined.

This was particularly true for ‘responsive’ projects that required just-in-time skill acquisition. These projects shaped both what skills were needed and how teams were assembled and helped to build the shared repertoire of tools and methodologic approaches for simulation design and delivery.

#### Partnerships beyond simulation programs

Building capacity also enabled, and was in turn enabled by, external partnerships, especially those between educational and health service institutions.

*“Interestingly*,* as our simulation fellows completed the course*,* there has also been capacity building within our local health networks as a secondary benefit of the course. … they return to their clinical environment with the capacity to promote and deliver translational simulation programs*” (Simulation Program leader, AHS)

Program leaders recognised that they were often pioneers of this work and were likely to build further capacity by supporting others in their simulation or health service networks.

*“We have also reached out beyond SCHN to other [statewide] Health Pillars who are interested in what we are doing and what we have developed. I hope we will work with them to develop a statewide program of* [translational simulation] *to embed across [the state].*” (Simulation Program leader, SCHN)

## Discussion

**Fig. 2:**
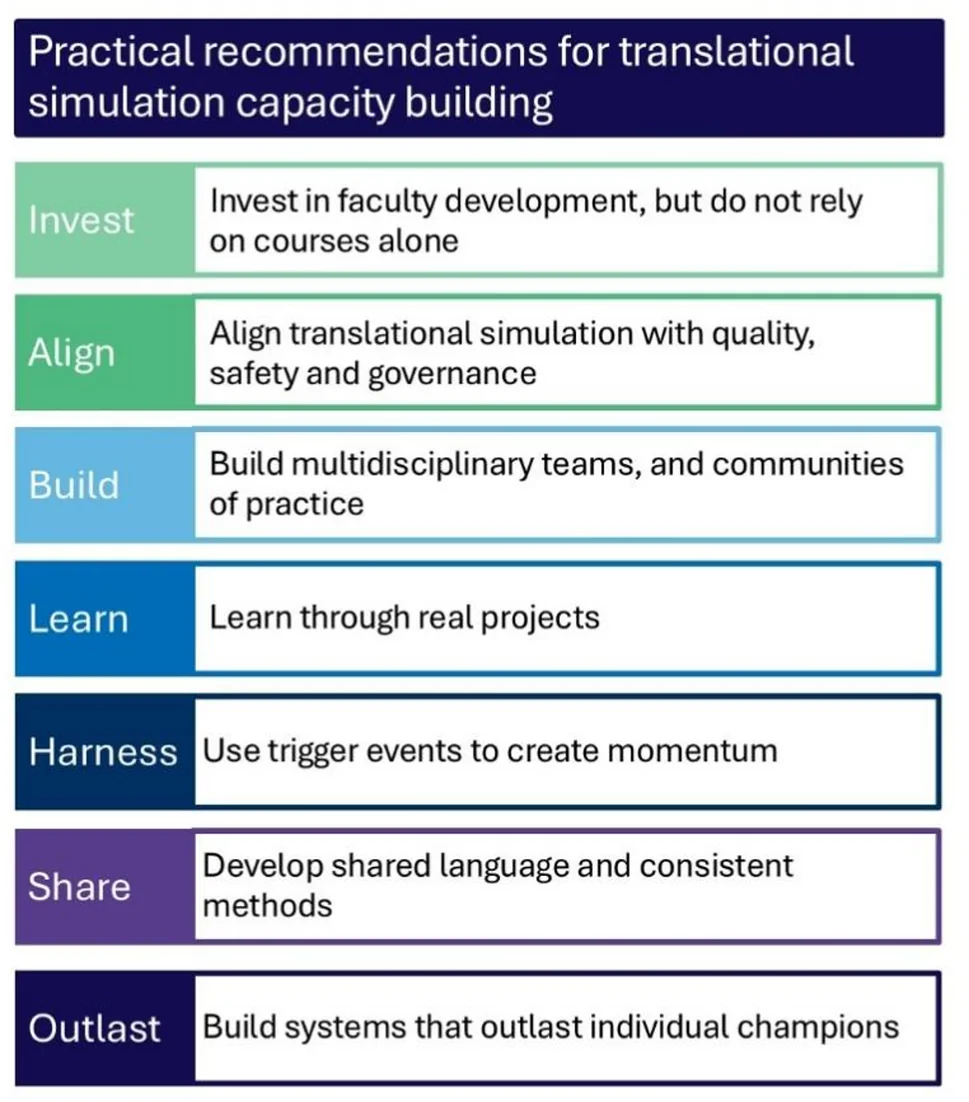
Practical recommendations for translational simulation capacity building

Translational simulation capacity is not built by training faculty first and then expecting programs to follow. Our findings suggest programs must create (or find) conditions in which translational simulation can become integrated and operationally aligned to improve health care quality and safety (Fig. 2). Clear strategy, effective leadership, mature operational processes, and fostering relationships are all crucial. In this discussion we focus on when and how faculty development is helpful within these broader contextual influences on capacity building.

Simulation programs - and the individuals who participate in faculty development from those programs - need to be ready to use the knowledge and skills gained in faculty development. In our study, the timing of participation in the ATS course was influenced by upcoming challenges and opportunities for their simulation programs. These trigger events provided motivation and unlocked resources at an institutional level. Focus on ‘real work’ during the course fostered engagement by learners, and naturally led to peer collaboration on projects contextually relevant to the institution. Our three programs were well established within their institutions and had sufficient operational sophistication to support staff members to use the knowledge and skills gained in the ATS course. The exact point at which organisations are ‘ready’ for translational simulation faculty development remains a grey area. But the signal from our study is that there is a tight coupling; translational simulation faculty development improves translational simulation capacity, but adequate capacity (e.g. mindset, resources, leadership) needs to exist before faculty development can be most helpful.

Krogh and McNaughton articulated a similar concern about *educationally focused* simulation faculty development [24]. They questioned the value of brief workshop formats for simulation debriefing and reflected on “*…a naïve belief about our learners’ abilities to translate lessons learned in courses to their day-to-day practice*” [24]. We support their contention, and do not blame individuals for this lack of translation, but rather a lack of alignment with the contextual realities in which knowledge and skills must be applied. This may point to an important distinction for translational simulation: faculty development may need to be theorised differently from educationally focused simulation faculty development. The unit of change is not only the individual educator, but the simulation program situated within its organisational system.

Decisions to participate in a *group-based* faculty development recognise the critical relational factors that support learning and work in translational simulation. Training teams, rather than individuals, builds organisational knowledge and the relationships needed to harness them. A shared understanding of the nature of translational simulation, and it’s value and positioning within an organisation, supports effective, collaborative work that is aligned with organisational priorities. This effectively draws together the faculty development community and the workplace community, as described O’Sullivan and Irby [21]. Our experiences in this study and beyond have reinforced the decision to offer the ATS course within the ‘formal, group’ quadrant of Steinart’s faculty development model [20].

Absent from our analysis was any extensive reflection on the *content* of the ATS course. This is in juxtaposition with much of the literature on simulation faculty development. Our research aim was not to evaluate the course, and we make no claim as to the superiority or otherwise of its content. That said, our themes’ powerful focus on context validate our inclusion of implementation science, change management, and demonstrating value in the final module of the course.

In a similar vein, nor do we suggest that the three programs we explored are exemplars, particularly in an emerging field in which characteristics of successful translational simulation programs are still being explored. The program leaders in our author team were at least partly motivated to participate by the opportunity to reflect on their respective strengths and weaknesses, and to compare approaches to faculty development and capacity building.

We identified several trade-offs within translational simulation capacity building. Established commitments to educational simulation can limit the extent to which individuals and programs can apply what was learned through a course. The reframing required of simulation teams is not always seamless. This underlines the importance of leadership in clarifying mission, supporting staff through change, and maintaining focus on the distinct mission of their programs.

### Limitations

We acknowledge limitations in our study. Data were drawn exclusively from an Australian context. The institutional and cultural conditions of this geographical and socio-political context will inevitably have shaped the narratives and our subsequent analysis. The participating programs were also relatively established, led by individuals with a strong interest in translational simulation, and sufficiently resourced to enrol staff in a formal faculty development course. The findings may therefore be most transferable to comparable programs seeking to deepen or consolidate translational simulation capacity, rather than to early-stage, under-resourced or differently governed programs. However, our cautious advice to less established programs is similar – to invest in faculty development only in parallel with broader progress on building capacity for translational simulation. It may be that alternative professional development experiences such as short sabbaticals within established programs can provide tacit and explicit lessons in building capacity may be embedded in the experience.

The data comprised structured narrative accounts rather than direct observation, interviews with a broader stakeholder group, organisational documents or outcome data. The findings therefore reflect how program leaders and course faculty interpreted and represented their experiences of capacity building. Perspectives from executives, frontline clinicians, quality and safety partners, and course participants beyond the author group were not included.

A significant reflexive challenge arose from the research team analysing narratives drawn from our own experiences. This insider position provided access to rich contextual detail, shared language and trust, but also created a risk that familiar interpretations would appear self-evident. We sought to mitigate this through ongoing reflexive team discussion, deliberate attention to alternative readings, and explicit acknowledgement of our dual roles as educators, program leaders and researchers. We do not claim to have analysed the data from a neutral standpoint, and recognise that other interpretations are possible.

## Conclusion

Building capacity for translational simulation is much more than training individuals. It requires strategy, leadership, governance, relationships, and operational conditions that allow translational simulation to be practiced effectively. In our programs, faculty development was most helpful when aligned with organisational priorities, undertaken collectively, and connected to real projects, mentorship, and opportunities for ongoing practice. Faculty development is therefore best understood as one element of a broader institutional strategy for translational simulation capacity building.

## Supplementary Information

Supplementary Material 1.

## Data Availability

Datasets (written narratives) are available on reasonable request.

## Abbreviations

AHS: Adelaide Health Simulation
AI: Artificial intelligence
ASPiH: Association for Simulated Practice in Healthcare
ATS: Applied Translational Simulation
BAU: Business as usual
CoP: Community of practice
ED: Emergency department
HPE: Health professions education
QI: Quality improvement
ROI: Return on investment
RTA: Reflexive thematic analysis
SCHN: Sydney Children’s Hospital Network
WH: Western Health
