# Supplementary material for: Building capacity for translational simulation: the role of faculty development
Source: Adv Simul (Lond). 2026 Aug 10;11:85. doi: 10.1186/s41077-026-00470-7 (PMC13746632; doi:10.1186/s41077-026-00470-7)
Supplement: Supplementary file 1 [file 41077_2026_470_MOESM1_ESM.docx]

# Supplementary Material

### Building capacity for translational simulation –

### Prompts for narrative reflection

- Describe the mission, scope of your simulation program
- Give us a picture of an average week in your sim program – types and numbers of simulations and participants.
- What part of your work would you describe as translational simulation. Can you give some examples?
- Tell us about your simulation program faculty – what backgrounds and professions? Balance of FT/ PT/ casual
- Describe your approach to simulation faculty development within your program?
- Structured versus informal?
- Group versus individual?
- What content areas - debriefing, giving feedback, technical aspects of simulation delivery, curriculum design and educational theory
- How do these elements interact - mentoring and coaching; relationships and networks; organizations, systems, and cultures; and tasks and activities
- How are faculty recruited/ prepared/ support for their role in translational simulation delivery?
- What do you think are the key knowledge and skills for faculty you involve (or would hope to involve) in translational simulation

(*Prompt – as compared to list in Brazil et al Faculty development for translational sim Table 1* <https://advancesinsimulation.biomedcentral.com/articles/10.1186/s41077-023-00265-0/tables/1>)

- How is your response influenced by your program context?
- What were your motivations for enrolling your team in the ATS subject?
- How has that contributed to building capacity for translational simulation in your program context?
- What barriers are there to building capacity within your faculty team for translational simulation?
- What future plans do your have for building capacity within your faculty team for translational simulation?
